# One Assay, Nine Targets: Advancing Viral Surveillance with Multiplex RT-ddPCR

**DOI:** 10.1021/acs.analchem.5c04372

**Published:** 2025-10-03

**Authors:** Anastasia Zafeiriadou, Georgia Georgakopoulou, Foteini Pitaouli, Nikolaos Thomaidis, Athina Markou

**Affiliations:** Laboratory of Analytical Chemistry, Department of Chemistry, 68993 National and Kapodistrian University of Athens, Zografou, 15771, Athens, Greece

## Abstract

Viral infections continue to pose a major global health challenge, driven by factors such as population growth, migration, and environmental change, all of which contribute to the emergence and reemergence of infectious viruses. Advances in technology now enable the detection of multiple targets from a limited sample volume; however, few studies have fully leveraged these capabilities. In this study, we developed and analytically validated a highly sensitive and specific 9-plex one-step RT-ddPCR assay for the detection of high-risk viruses, including SARS-CoV-2 (N1 and N2 genes), Influenza A and B, Respiratory Syncytial Virus, Hepatitis A and E, along with both endogenous and exogenous controls. Initial validation was conducted using synthetic DNA, followed by application to 38 wastewater samples—complex and heterogeneous matrices that often harbor multiple viral targets. The assay demonstrated excellent analytical performance in terms of sensitivity, linearity, specificity, and reproducibility with detection limits ranging from 1.4 to 2.9 copies/μL depending on the viral target. A direct comparison with singleplex ddPCR assays revealed high concordance (Mann–Whitney test, *p* > 0.1), indicating no statistically significant differences and highlighting the efficiency of the multiplex format. To the best of our knowledge, this is the first study to simultaneously quantify nine targets in a single RT-ddPCR reaction. The developed assay shows a strong potential for application across various sample types, including wastewater.

## Graphical abstract

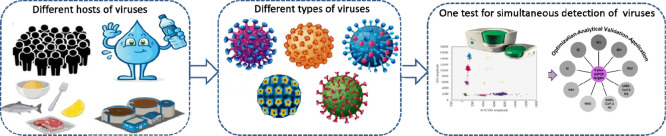

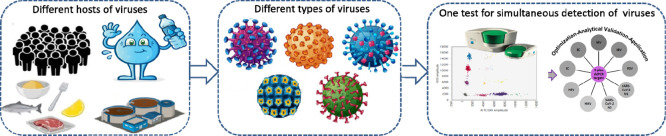

## Introduction

Viral infections remain a critical threat to global public health despite ever-increasing advances in healthcare. Population growth and migration, changing weather conditions and other social, biological, and environmental mechanisms often lead to the emergence of new species or re- emergence of once-controlled infections.^1^ Robust analytical and detection methods are necessary to detect their spread or disease outbreaks at an early stage. Severe Acute Respiratory Syndrome Virus 2 (SARS-CoV-2) has been the focus of interest since its emergence, but surveillance of other epidemiologically important viruses is also necessary to detect earlier unexpected trends in their seasonality or circulation. Influenza A (IAV) and B (IBV) viruses and respiratory syncytial virus (RSV) are widespread in communities, and their changing seasonal patterns and cocirculation in the post SARS-CoV-2 emergence era are not yet defined. Food-borne viruses, such as hepatitis A (HAV) and hepatitis E (HEV), are also considered important epidemiological targets, as they are emerging as agents of acute hepatitis worldwide^2,3^ and have become the focus in many surveillance programs.

Viruses can be isolated through various sources such as food and water,^4–9^ wastewater and clinical samples.^10–14^ Direct virus detection from any source without the need to introduce viral particles into a host cell line is a widely used method and is divided into two main categories; the nucleic acid amplification tests and immunoassay-based diagnostics.^15^ Of them, nucleic-acid based assays are highly specific and sensitive and are often considered as the gold standard methods for viral diagnostics.^16^ Quantitative polymerase chain reaction (qPCR) has been the mainstream detection method in viral diagnostics, but its reliance on a standard curve for quantification and the susceptibility to inhibition have recently drawn focus on the application of droplet digital PCR (ddPCR).The potential of ddPCR due to the advantage of absolute quantification and higher inhibitor-tolerance has been demonstrated in many applications, especially in environmental samples that are characterized by complex matrices^17,18^ and clinical samples.^19–21^ Thanks to improvements in ddPCR technology, researchers can now also use advanced multiplexing technology that enables the simultaneous detection of multiple targets in a single reaction and reduces technical errors, the reagent and time needs.^22^

ddPCR technologies take advantage of the different fluorescence channels that can be used in droplet readout in order to detect multiple targets. The majority of ddPCR assays utilized a 2-color channel ddPCR system that is able to detect two targets simultaneously^23,24^ and have extended its multiplexing capabilities by mixing different concentrations of primers and probes to differentiate and identify more targets on a 2-D amplitude plot. This method has been used in viral diagnostics and has made possible the detection of three to five targets in a single ddPCR reaction.^21,25,26^ However, this approach is usually complicated and difficult to apply in complex matrices that have a higher number of inhibitors and often decrease the specificity of the assay. Newer ddPCR technologies have 6- or 7-color channels and can detect four,^27,28^ five,^29^ or six^30^ targets in a single run. This multiplexing capacity is not yet extensively used, and more research should be done in this field to fully utilize this method, especially in the context of viral diagnostics. Up to date, various multiplex RT-ddPCR assays have been developed and evaluated for the detection and quantification of respiratory^28–33^ and hepatitis viruses^24,34–37^ that were isolated from variable sources. SARS-CoV-2, Influenza, and RSV viruses pose a major public health problem and are a leading cause of morbidity and mortality worldwide, while Hepatitis A and E viruses lead to severe hepatitis infections in both developed and developing countries,^38,39^ raising the need for developing more accurate and time-saving assays for the detection and quantification of these pathogens.

In the present study, a novel, sensitive and specific one-step 9-plex RT-ddPCR assay was developed and analytically validated for the simultaneous detection of high-risk contracting viruses such as SARS-CoV-2 (N1, N2), Influenza A, B, RSV, Hepatitis A and E and one endogenous and one exogenous control. The developed assay was further applied to 38 wastewater samples that were collected from the Attica region, to evaluate its performance in highly complex and heterogeneous matrices. Wastewater is very likely to harbor multiple viruses simultaneously, since it reflects the health status of the entire community tested, highlighting the need for developing highly sensitive and specific multiplex assays. To the best of our knowledge, this is the first study of the simultaneous absolute quantification of nine targets in a single reaction.

## Materials and Methods

### Wastewater Sampling

24-h composite flow proportional raw wastewater samples were collected at the Wastewater Treatment Plant (WWTP) of Attica, the region of Greece that includes the greater Athens area and its suburbs. 38 raw wastewater samples were collected from December 2023 to February 2025 and followed a concentration and extraction protocol that has been previously validated.^40,41^ Briefly, for nucleic acid extraction, the Enviro Wastewater TNA Kit (Promega, United States) was used according to the manufacturer’s instructions, starting with 40 mL of wastewater, which was further concentrated to 1 mL. Total nucleic acids were then extracted and eluted in a final volume of 100 μL. This direct capture-based method has been previously evaluated against various concentration techniques and was found to be the most effective in terms of recovery, processing time, and cost.^40,41^ Wastewater samples were collected in precleaned 1 L high-density polyethylene bottles and transported to the laboratory at 4 °C, where they were processed immediately upon arrival. Biosafety guidelines were followed during sample collection, transport, and analysis.

### Multiplex One-Step RT-ddPCR

A one-step 9-plex RT-ddPCR assay was developed and analytically validated for the simultaneous detection and absolute quantification of (a) seven viral targets; SARS-CoV-2 N1 and N2 genes (nucleocapsid proteins), Influenza A M gene (matrix protein), Influenza B NS gene (nonstructural protein), RSV M gene (matrix protein), Hepatitis A virus 5′UTR gene (5′-untranslated region) and Hepatitis virus E ORF3 gene (Open Reading Frame 3 protein), (b) Beta-2 microglobulin (B2M) as an endogenous internal control (IC), and (c) a synthetic DNA oligo as an external control (EC). All primers were *in silico* designed in specific conserved regions of the viral genomes. The selection of two regions of SARS-CoV-2 reduces the probability of false negative results resulting from accumulating of genetic alterations in the viral genome, which can reduce test sensitivity and detection rates.^42,43^ The inclusion of the IC and EC controls in the ddPCR assesses accurate sampling and RT-ddPCR performance, while helping to limit the number of false negative results.^44^ All hydrolysis probes were designed with a 6-carboxyfluorescein (FAM), hexachlorofluorescein (HEX), rhodamine X (ROX), cyanine 5 (Cy5) or ATTO590 fluorophore and ZEN/Iowa Black quenchers for more efficient quenching (Integrated DNA Technologies, USA) (Table S1, primer sequences). Multiplex one-step RT-ddPCR was performed in the QX600 Droplet Digital PCR System (Bio-Rad, USA). At first, primer/probe sets for SARS-CoV-2 N1, IAV, IBV and HAV were prepared at a final concentration of 900 nM/300 nM. These are characterized as the high targets due to the higher fluorescence signal (ppmix A). For RSV, HEV, and the EC, the primer/probe sets were prepared at a final concentration of 400 nM/100 nM, while for SARS-CoV-2 N2 and B2M, the primer/probe sets were prepared at a final concentration of 450 nM/150 nM (ppmix B). These are characterized as the low targets due to the lower fluorescence signal in the corresponding channel. By adding one FAM, HEX, ROX and ATTO590 assay at 1× concentration and the other assay at a lower concentration, an upper and a lower cluster can be formed in the same well, resulting in clearly separated clusters in each channel in a 2D scatter plot, allowing multiplexing of the developed assay.

The developed ddPCR assay was performed using One-step RT-ddPCR Advanced kit for Probes (Bio-Rad, USA) and the reaction mix consisted of 5.0 μL of Supermix, 2.0 μL of Reverse Transcriptase, 1.0 μL of 300 mM dithiothreitol (DTT), primers and probes at their optimized final concentrations (Figure 1), 5 μL of RNA template, and H_2_O to a final volume of 20 μL. PCR was performed in the C1000 Touch Thermal Cycler (50 °C/1h for the reverse transcription step, 95 °C/10 min, 40 cycles of 94 °C/30 s and 61 °C/1 min and a final step at 98 °C/10 min). A temperature ramp rate of 2 °C/s was set on all PCR steps. 96-well plate was read in the QX600 Droplet Reader (Bio-Rad, USA) and the absolute copy number of the nine targets was calculated using the QuantaSoft analysis software (Bio-Rad, USA), according to the Poisson Distribution. Wells that had <10000 droplets, were excluded from the analysis. Positive and negative controls were used in each RT-ddPCR run to evaluate the performance of the assay.

**Figure 1:**
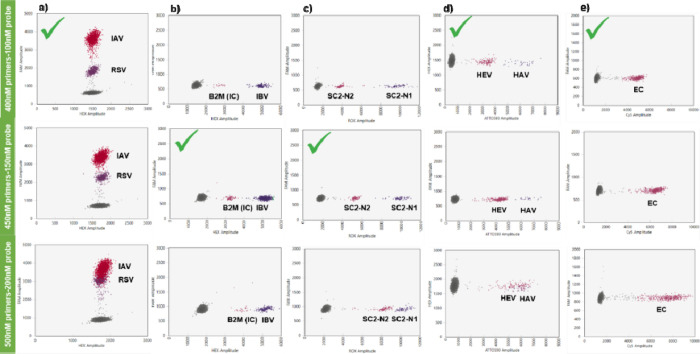
Different concentrations of the low fluorescence targets tested for (a) IAV and RSV, (b) IBV and B2M-IC, (c) N1 and N2 SARS-CoV-2 genes (SC2-N1 and SC2-N2), (d) HAV and HEV, and (e) EC. The check symbol marks the best option for each target.

### DNA and RNA Standards

Six synthetic DNA oligonucleotides were developed and used for the analytical validation of the assay (gBlocks; Integrated DNA Technologies, USA; Table S2). Each of these synthetic oligonucleotides contained the target sequences of IAV (106 bp), IBV (103 bp), RSV (83 bp), HAV (167 bp) and HEV (89 bp) including primer and probe binding sites. Each synthetic DNA oligo was diluted with Tris–EDTA (TE) buffer to obtain a stock solution with a concentration of 10 ng/μL.

EURM-19 synthetic single-stranded RNA standard (European Commission, Joint Research Centre, Geel, Belgium) that contains fragments of SARS-CoV-2 regions was used for the analytical validation of N1 and N2 genes.

For the analytical validation of B2M as an internal control, RNA derived from the MCF-7 cell line was used. B2M gene mRNA is commonly employed as an endogenous control because it is expressed in all nucleated cells and serves as a human biomarker with good stability and minimal degradation over 24-h in wastewater samples.^45,46^ The designed synthetic oligo that was used as external controls was a 128 bp DNA sequence that does not align to the human or tested viral genomes (Table S2). External and internal controls can help assess the performance of the assay, validate the results and provide a reference for interpreting data. External control ensures that the assay is functioning as expected and that data can be interpreted with confidence.

## Results

### Optimization of One-Step Multiplex RT-ddPCR

#### Annealing Temperature and Time

Optimization of the annealing temperature is critical for the reaction’s specificity. To optimize the annealing temperature, the multiplex RT-ddPCR assay was performed at different temperatures that ranged from 58 to 62 °C. 61 °C was chosen as the best option for all targets, based on better separation of clusters, highest specific signals for all channels, and minimal amounts of “rain” for each virus type. Modest adjustments to the standard ddPCR cycling parameters, such as reducing the ramp rate at 1 °C, increasing the annealing/extension time to 2 min or increasing the number of cycles, may also have positive influence on the cluster separation.^47^ Apart from the annealing temperature, it was found that the optimal annealing time was 1 min, compared to the 45 and 2 min tested in the study and discarded due to poorer cluster separation. Subsequent ddPCR experiments were performed using the optimal conditions for the annealing step (61 °C/1 min) (data not shown).

#### Concentration of Primers and Probes

The QX600 Droplet Reader system offers the ability of detecting up to 12 targets, by detecting two different levels of fluorescence amplitude in each of the 6-color channels. Bio-Rad instructor’s manual suggests that higher amplitude targets should be prepared at a final concentration of 900 nM/250 nM (1×) of primers/probes and that the lower amplitude targets at 0.5×, respectively. To further improve the cluster separation of the different targets within the same fluorescence channel, (a) 400 nM–100 nM, (b) 450 nΜ–150 nM, and (c) 500 nΜ–200 nM concentrations of primers/probes were tested, while the higher amplitude targets remained at the final concentration of 900 nΜ–300nΜ. The optimum concentrations for each channel were selected, based on the best separation from the background (negative droplets), the clear separation of the clusters and the less “rain” drop, since these are important decisive factors that influence the specificity of the assay (Figure 1). According to our results, the best option for RSV, HEV and EC was at 400 nM–100 nM primers/probes, while the 450 nΜ–150 nM primers/probes mix was the optimum concentration for B2M (IC) and SARC-CoV-2N2 targets.

### Analytical Validation

Synthetic oligonucleotides for each virus type (gBlocks; Integrated DNA Technologies, USA), the single-stranded RNA EURM-019 (European Commission, Joint Research Centre, Geel, Belgium), and RNA derived from the MCF-7 cell line, where only B2M is expressed, were used for the preparation of the controls. The analytical specificity and the analytical sensitivity in terms of the limit of detection (LOD) and limit of quantification (LOQ), the linear dynamic range (LDR) and the intra- and interassay repeatability of the assay were estimated.

#### Analytical Specificity

We checked the analytical specificities of the primers and probes that were designed and used for multiplex PCR and assessed analytical specificity when only one target was used as a template. For the evaluation of the analytical specificity of the assay, eight specific controls were used, as clearly defined above, at a final concentration of 100 copies/μL. According to our results, we did not observe any of the nonspecific interactions between the eight oligonucleotides used, and the analytical specificity was excellent. Only one specific droplet cluster was obtained in the expected fluorescence channel, while the other four channels were characterized by the absence of fluorescent signal (Figure 2); therefore, we concluded that the assay was able to discriminate the presence of virus specifically for each target.

**Figure 2:**
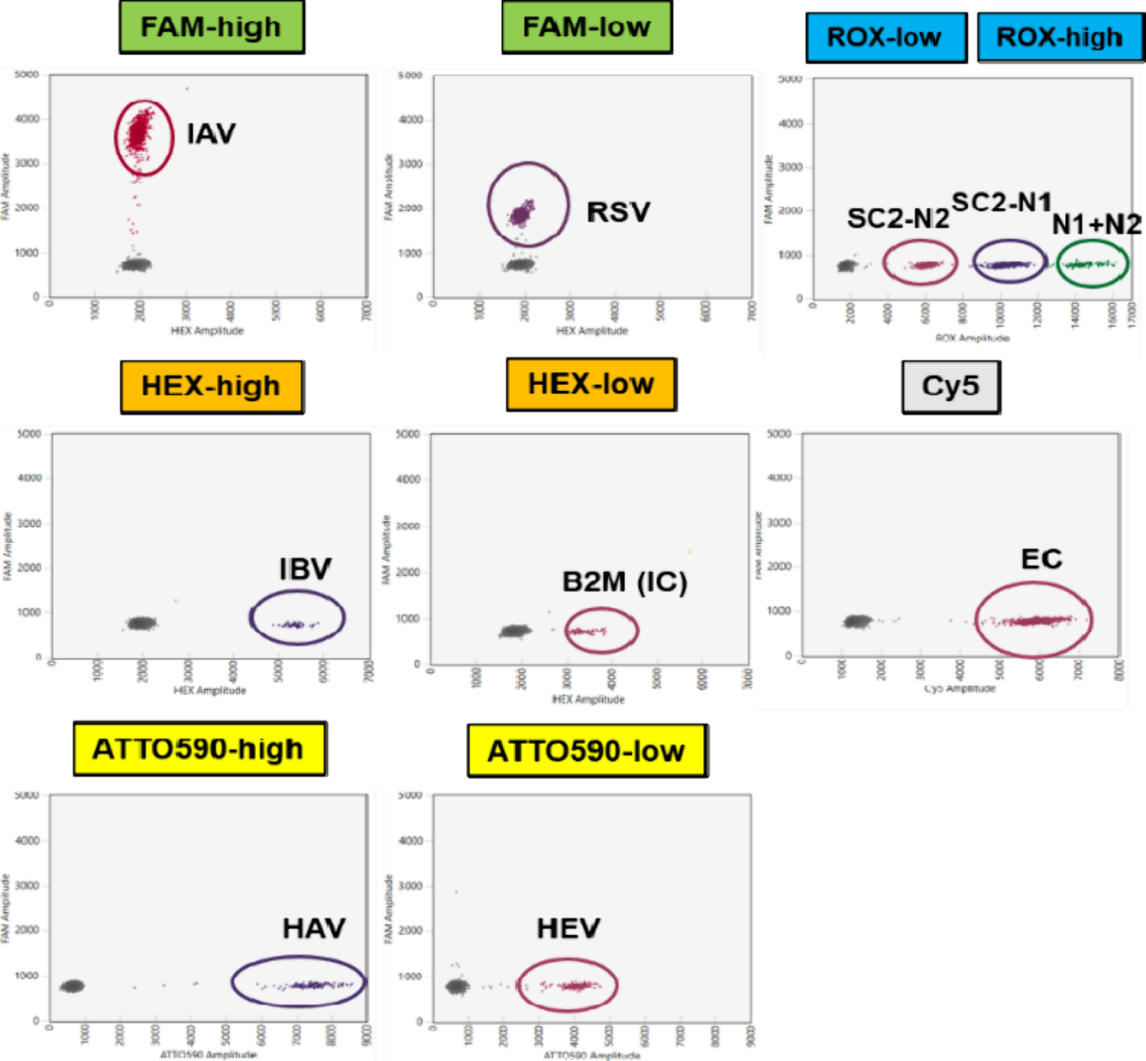
Analytical specificity of the developed one-step multiplex RT-ddPCR assay. 2-D plots representing each target in the individual assays.

#### Comparison between Singleplex and Multiplex ddPCR

Newly developed multiplex assays should be carefully optimized to maintain—or even enhance—the analytical sensitivity of their singleplex counterparts. In this study, viral copies at varying concentrations were analyzed using a multiplex RT-ddPCR assay and compared to individual singleplex assays targeting each virus as well as the respective quality controls. All standards were tested in triplicate, and the Mann–Whitney test was used to assess the statistical differences between groups. The results indicated that the multiplex and singleplex RT-ddPCR assays produced comparable quantitative outcomes, with no statistically significant differences observed (*p*-values > 0.05) (Figure 3, Table S3).

**Figure 3:**
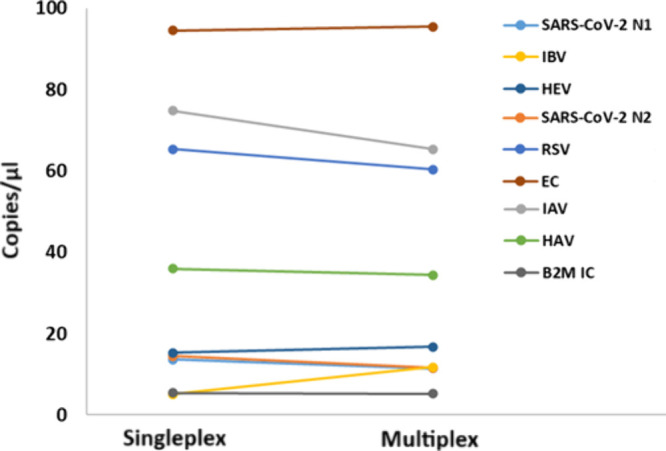
Multiplex to singleplex comparison. Determination of copies/μL of each virus using both multiplex and singleplex PCR for the evaluation of the effect of multiplexing assay.

### Analytical Sensitivity

For the limit of detection (LOD) evaluation, synthetic oligonucleotides corresponding to each target virus were tested at three concentration levels: CAL1 (high), CAL2 (intermediate), and CAL3 (low). CAL1 was prepared at 200 copies/μL for all targets. CAL2 and CAL3 were generated through 10-fold and 100-fold serial dilutions of CAL1, respectively. CAL1 and CAL2 samples were analyzed six times, while CAL3 was assessed in 20 replicates. The LOD was estimated using the standard deviation (SD) of CAL3 measurements and defined as the mean value ± 2 × SD, corresponding to a 95% Confidence Interval (CI). The LOD value was set at 2.2 (95% CI: 1.7–2.7), 2.0 (95% CI: 1.6–2.4), 1.4 (95% CI: 1.1–1.7), 2.1 (95% CI: 1.7–2.4), 2.9 (95% CI: 2.4–3.3), 1.8 (95% CI: 1.4–2.2) and 1.9 (95% CI: 1.6–2.2) copies/μL of sample input for SARS-CoV-2 N1 gene, N2 gene, IAV, IBV, RSV, HAV, and HEV, respectively (Table S4).

LOQ was set as the lowest detected concentration that had a coefficient of variation (CV) ≤ 25 and was set at 5.8, 6.1, 3.46, 5.45, 5.89, 6.14, and 4.61 copies/μL for SARS-CoV-2 N1 gene, N2 gene, IAV, IBV, RSV, HAV, and HEV, respectively (Table S4).

The detection rate was 100% at all concentration levels for SARS-CoV-2, IBV, HEV, B2M, and EC. At the lowest concentration level, IAV and HAV were detected in 18 out of 20 replicates, while RSV was detected in 19 out of 20.

#### Intra- and Interassay Repeatability

Intra-assay repeatability was evaluated by the same standards that were used to evaluate the LOD and LOQ of the developed RT-ddPCR assay (Table 1). CV% ranged from 7.18 to 46.1% for SARS-CoV-2 N1 and 6.77 to 41.7% for SARS-CoV-2 N2 gene, 6.75 to 43.7% for IAV, 5.57 to 38.1% for IBV, 4.43 to 31.1% for RSV, 4.75 to 40.8% for HAV, and 4.42 to 31.4% for HEV.

**Table 1:** Intra-assay Repeatability of the Newly Developed Assay

Virus Type		Average copies (μL)	SD	CV%
SARS-CoV-2 N1	CAL1	66.3	4.78	7.18
	CAL2	5.80	1.12	7.18
	CAL3	0.55	0.25	46.1
SARS-CoV-2 N2	CAL1	69.56	4.71	6.77
	CAL2	6.08	1.18	19.5
	CAL3	0.50	0.21	41.7
IAV	CAL1	40.21	2.71	6.75
	CAL2	3.46	0.80	23.2
	CAL3	0.36	0.16	43.7
IBV	CAL1	72.79	4.05	5.57
	CAL2	5.45	0.69	12.6
	CAL3	0.51	0.20	38.1
RSV	CAL1	69.94	3.10	4.43
	CAL2	5.89	0.52	8.87
	CAL3	0.72	0.22	31.1
HAV	CAL1	63.29	3.01	4.75
	CAL2	6.14	0.83	13.5
	CAL3	0.45	0.18	40.8
HEV	CAL1	66.90	2.96	4.42
	CAL2	4.61	0.45	9.84
	CAL3	0.48	0.15	31.4

Reproducibility or interassay repeatability was evaluated by analyzing a positive control that contained all targets, in nine separate RT-ddPCR runs on nine different days. The CV% was ≤ 25 for all targets (Table 2). The use of one positive control is more practical and efficient and provides a reliable measure of method performance across runs.

**Table 2:** Inter-assay Repeatability of the Newly Developed Assay

Virus Type	Average copies (μL)	SD	CV%
SARS-CoV-2 N1	26.2	4.88	18.6
SARS-CoV-2 N2	27.7	5.41	19.5
IAV	58.7	4.44	7.6
IBV	16.4	3.2	19.4
RSV	64.3	5.31	8.3
HAV	40.4	4.48	11.1
HEV	23	2.51	10.9
B2M (IC)	14.7	3.50	23.7
EC	82.3	7.1	8.6

#### Linear Dynamic Range

The linear dynamic range was evaluated using CAL1, CAL2 and CAL3, which correspond to five different concentration levels, as mentioned above. The results are presented in a linear regression plot showing the mean value of absolute copies/μL of sample input (*Y*-axis) against the dilution factor (*X*-axis) (Figure S1). According to our results, the correlation coefficients (*R* ^2^) ranged from 0.9991 to 0.9999, indicating a precise linear relationship.

## Application of the Newly Developed One-Step Multiplex RT-ddPCR Assay in Wastewater Samples

After analytical validation, the developed assay was used to detect and quantify the target viruses in 38 raw wastewater samples from the Attica region. Wastewater sampling is not just limited to one virus. It is a broad tool for detecting various viruses, including enteric viruses, respiratory viruses (like influenza and SARS-CoV-2), and hepatitis viruses (Figure S2). This versatility makes it valuable for monitoring different types of viral diseases that can impact public health.

Despite the difficult matrix of wastewater samples and the influence of inhibitors, the IC was detected and was stable in all samples (mean value; 1.4 × 10^6^ copies/L), verifying the accuracy of the analytical process in each sample. In this study, a synthetic DNA sequence that is not naturally present in the sample was used as an external control. In each reaction, 700 copies/μL of external control were spiked to monitor the performance of the developed one-step ddPCR assay to detect problems related to the droplet generator, ddPCR reagents, and the QX600 Droplet reader. According to our results, the external control was detected in all samples with the mean recovery rate of 83.1 ± 12.3 and the CV% was 14.8%.

According to the viruses’ detection, 38/38 (100%), 28/38 (73.7%), 28/38 (73.7%), 25/38 (65.8%), 0/38, and 0/38 samples were positive for SARS-CoV-2, IAV, IBV, RSV, HEV and HAV transcripts, respectively (Table 3). For the quantification of SARS-CoV-2, the average absolute copy numbers of N1 and N2 transcripts were used as similar copy numbers were found in both regions, indicating equal efficiency and comparable copies/μL (Table S5). The logarithmic scale of viral load provides a more accurate representation of our results (Figure 4)

**Figure 4:**
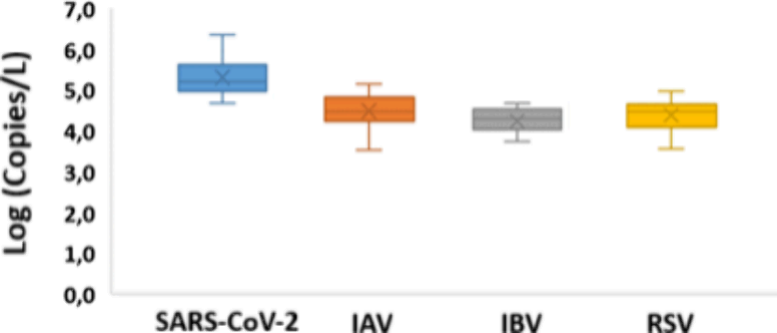
Boxplots and T-Whiskers of SARS-CoV-2, IAV, IBV, and RSV expressed in log (copies/L). The lower and upper boxes denote 25th and 75th percentiles.

**Table 3:** Range and Mean Value of Viral Copies/L per Each Target

Virus Type	Range of viral load (copies/L)	Mean value (copies/L)
SARS-CoV-2	4.8 × 10^4^–2.3 × 10^6^	3.5 × 10^5^
IAV	3.3 × 10^3^–8.6 × 10^4^	4.3 × 10^4^
IBV	5.4 × 10^3^–4.6 × 10^4^	2.1 × 10^4^
RSV	3.6 × 10^3^–9.3 × 10^4^	3.3 × 10^4^

The viral load ranged from lower to higher numbers for each virus type and between the different types. Respiratory viruses have different seasonal patterns, so different viral loads and prevalence can be expected throughout the year.^48^ The developed assay is able to detect multiple types of Influenza A that circulate in communities (H1N1, H3N2, H1N2, H7N9, and H9N2); however, Influenza A positive cases are most likely linked to subtypes H1N1 and H3N2 that are the most prevalent and responsible for the vast majority of seasonal Influenza epidemics each year. Additionally, both subtypes of Influenza B (B/Yamagata, B/Victoria) and RSV (RSV A and B) that commonly circulate among humans annually are amplified by the developed assay, contributing to the positive cases found in samples.

## Discussion

ddPCR is a relatively recent molecular technique that offers several advantages over conventional PCR methods. These include higher sensitivity and precision, greater tolerance to inhibitors and absolute quantification without the need for a standard curve.^49–51^ As a result, ddPCR has seen growing application in the detection and quantification of viruses across various types of clinical and environmental samples.^52^ It is also increasingly being used as a complementary tool to next-generation sequencing (NGS) technologies.^53^

Viral infections remain a significant global public health concern, contributing to an increased morbidity and mortality worldwide. In addition to the recent COVID-19 pandemic—which placed unprecedented strain on healthcare systems—seasonal respiratory viruses such as Influenza A, Influenza B, and Respiratory Syncytial Virus (RSV) continue to pose serious challenges due to their annual resurgence and cocirculation. These viruses not only threaten public health but also disrupt immunization programs and vaccine effectiveness.

Although multiplex qPCR assays^54–56^ are widely used for viral detection, there are relatively fewer multiplex ddPCR assays available.^21,31,57^ This is primarily due to the limitations of most commonly used ddPCR systems, which typically offer only two fluorescent detection channels, thereby restricting the number of targets that can be simultaneously detected in a single reaction.^24,26^ To overcome these limitations, we aimed to develop and validate a 9-plex ddPCR assay capable of simultaneously detecting seven viruses and two internal quality control targets within a single reaction.

Initially, the 9-plex ddPCR assay was evaluated for its performance. The LOD ranged from 1.4 to 2.9 copies/μL of sample input (0.36 to 0.72 copies/μL measured in ddPCR) and the clear separation between droplet clusters demonstrated the assay’s high sensitivity and specificity. The inclusion of internal and external quality controls was critical for verifying the reliability of the results and detecting any issues throughout the analytical workflow. These controls were consistently detected in 100% of the experiments, reinforcing the robustness of the assay. The performance of the multiplex assay was comprehensively assessed and compared to conventional single-target ddPCR assays. When applied to wastewater samples, the assay revealed the presence of four or more viral targets in the majority of the tested samples. The higher positivity rate of respiratory viruses compared to hepatitis viruses in wastewater samples may be explained as respiratory viral infections are typically more prevalent in the general population and exhibit strong seasonal dynamics, leading to higher and more consistent wastewater signals. Moreover, respiratory viruses such as SARS-CoV-2, influenza, and RSV are shed at high titers in respiratory secretions (e.g., sputum, mucus, and saliva), which can readily enter wastewater systems, in addition to their occasional shedding in feces. In contrast, HAV and HEV endemicity is generally low in developed countries,^58^ largely due to improvements in water, sanitation, and hygiene (WASH) infrastructure, healthcare systems, and vaccination programs. Although HAV and HEV are known to be environmentally stable, their fecal shedding is intermittent, and community prevalence remains relatively low. Consequently, HAV/HEV RNA concentrations in wastewater often fall below the assay’s detection threshold, despite the viruses themselves being intact in the environment.

Recent studies have developed ddPCR assays reporting LODs ranging from 4.0 to 6.4 copies/reaction for SARS-CoV-2,^26,59^ with multiplex ddPCR assays achieving sensitivities of approximately 0.65 to 0.78 copies/μL for SARS-CoV-2, IAV, IBV and RSV.^33^ For enteric viruses, ddPCR assays have reported LODs of 5 to 6.1 copies/reaction for HAV^37,60^ and 1.8 copies/μL for HEV^61^ in singleplex and 12.6 and 8.9 copies/reaction for HAV and HEV, respectively, in duplex ddPCR assays.^62^ Here, we demonstrate that our newly developed assay can detect 7.2 to 14.4 copies/reaction (of ddPCR) for all tested targets, comparable to previously published assays, which targeted fewer targets per reaction.

Some researchers have developed multiplex ddPCR methods using standard two-color systems by varying the concentrations of primers and probes to distinguish multiple targets within the same channel,^63–65^ however this strategy is often complex. It is particularly challenging to implement in samples with PCR inhibitors, such as wastewater, as this complexity can reduce fluorescent signal resolution and impair target discrimination, ultimately limiting the assay’s utility for high-throughput applications. Recently, next-generation ddPCR platforms equipped with 6- or 7-color detection channels have been introduced to expand multiplexing capacity. The multiplexing capability of ddPCR enables simultaneous detection of multiple targets in a single reaction, reducing reagent use, sample processing time, and overall cost per analysis. Additionally, ddPCR offers absolute quantification without standard curves and shows high tolerance to inhibitors, enhancing accuracy and reducing the need for repeat testing—further supporting its cost-effectiveness. Malla et al. developed 5-plex^29^ and 6-plex^30^ assays targeting respiratory viruses and enteroviruses using multichannel systems. However, these studies did not incorporate internal or external controls within the same reaction, requiring separate reactions to verify the assay performance.

A potential limitation of our study is that the analytical specificity of our assays was evaluated against a limited panel of respiratory viruses. Given the extensive diversity of respiratory viruses, including additional viral strains could further strengthen specificity validation. However, the selected panel included representative and clinically relevant viruses from major respiratory virus families, and no cross-reactivity was observed. Future studies should aim to expand the validation panel as additional viral standards become available to further confirm the assay specificity. Moreover, another important limitation of the present study is that the assay was validated only on wastewater samples; future work will include testing on clinical specimens to further confirm its diagnostic applicability at the individual level. The current work demonstrates the feasibility of scaling multiplex detection from our earlier 4-plex assay to a 9-target panel. In future investigations, we plan to expand the panel further to cover a wider range of viral pathogens.

## Conclusions

The successful development of our 9-plex ddPCR assay for the simultaneous detection of respiratory and enteric viruses in wastewater represents a significant advancement in multiplex molecular diagnostics. This method offers a cost-effective, high-throughput, and efficient solution for public health surveillance by reducing reagent use, increasing testing capacity, and enabling real-time monitoring of pathogen prevalence in communities. Furthermore, the assay’s flexibility to detect multiple viruses in a single workflow makes it suitable for a wide range of sample types—including wastewater, clinical, and food matrices—and particularly valuable for addressing both emerging and re-emerging public health threats.

## Supplementary Material



## Notes

Additional details about the sequences of primers, probes, synthetic controls (Table S1, S2) and complementary details about the results section (Figure S1, S2, Table S3, S4) (PDF)
