# Supplementary material for: One Assay, Nine Targets: Advancing Viral Surveillance with Multiplex RT-ddPCR
Source: Anal Chem. 2025 Oct 3;97(40):22229–37. doi: 10.1021/acs.analchem.5c04372 (PMC12529474; doi:10.1021/acs.analchem.5c04372)
Supplement: Supplementary file 1 [file ac5c04372_si_001.pdf]

**Supporting Information**

# One Assay, Nine Targets: Advancing Viral Surveillance with Multiplex RT-ddPCR

Anastasia Zafeiriadou[1], Georgia Georgakopoulou[1], Foteini Pitaouli[1], Nikolaos Thomaidis[1] and Athina Markou[1*]

[1]Laboratory of Analytical Chemistry, Department of Chemistry, National and Kapodistrian University of Athens, Zografou, 15771, Athens, Greece.

**Corresponding Author**

***Athina Markou**, PhD

Assistant Professor of Analytical Chemistry

Laboratory of Analytical Chemistry

Dept of Chemistry, University of Athens, 15771

Athens, Greece

Email: atmarkou@chem.uoa.gr

Table of Contents

**Table S1.** Sequences of primers and probes of each target. ...............................................S3

**Table S2**. *In silico* designed synthetic controls that were used in the present study. .........S3

**Table S3.** Comparison of viral copies/µl of the newly developed multiplex RT-ddPCR assay and their corresponding singleplex assays. .........................................................................S4

**Table S4.** LOD and LOQ values obtained from the three concentration levels (CAL1, CAL2, CAL3) for the seven targets. ......................................................................................S4

**Table S5.** Copies/L of N1 and N2 targets of SARS-CoV-2 for each sample.........................S12

**Figure S1.** Linear dynamic range of the developed RT-ddPCR assay for the targeted viruses. SC2; SARS-CoV-2, IAV; Influenza A virus, IBV; Influenza B virus, RSV; Respiratory Syncytial Virus, HAV; Hepatitis A virus, HEV; Hepatitis E virus. .........................................S14

**Figure S2.** Representative RT-ddPCR dot plots from two wastewater samples. ...............S14

**References**..................................................................................................................S15

**Table S1.** Sequences of primers and probes of each target.

| Virus Type | Target Gene | | Sequence | Reporter | PCR product | References |
|---|---|---|---|---|---|---|
| **Influenza A** | M | F | 5’-caagaccaatcYtgtcacctctgac-3’ | - | 106bp | Modified from CDC[1] |
| | | R | 5’- gcattYtggacaaaVcgtctacg-3’ | - | | |
| | | Probe | 5’-tgcagtcctcgctcactgggcacg-3’ | FAM$^{high}$ | | |
| **Influenza B** | NS | F | 5’-tcctcaaYtcactcttcgagcg-3’ | - | 103bp | Modified from CDC[1] |
| | | R | 5’-cggtgctcttgaccaaattgg-3’ | - | | |
| | | Probe | 5’-ccaattcgagcagctgaaactgcggtg-3’ | HEX$^{high}$ | | |
| **RSV** | M | F | 5'-ggcaaatatggaaacatacgtga-3’ | - | 83bp | Modified from [2] |
| | | R | 5'-ctttttctaggacattgtattgaacag-3' | - | | |
| | | Probe | 5' -ctgtgtatgtggagccttcgtgaag-3' | FAM$^{low}$ | | |
| **SARS-CoV-2** | N1 gene | F | 5’-ggaccccaaaatcagcgaaat-3’ | - | 74bp | Modified from CDC [3] |
| | | R | 5’-ttctggttactgccagttgaatctg-3’ | - | | |
| | | Probe | 5’-caccccgcattacgtttggtggacc-3’ | ROX$^{high}$ | | |
| | N2 gene | F | 5’-gattacaaacattggccgcaaa-3’ | - | 71bp | Modified from CDC [3] |
| | | R | 5’-atgcgcgacattccgaagaa-3’ | - | | |
| | | Probe | 5’-cacaatttgcccccagcgcttcag-3’ | ROX$^{low}$ | | |
| **Hepatitis A** | 5’-UTR | F | 5'-tcaccgccgtttgcctag-3' | - | 167bp | |
| | | R | 5’-cctggaagaaagaagacagaaagc-3’ | - | | |
| | | Probe | 5’-cctgcaggttcagggttcttaaatctg-3’ | ATTO590$^{high}$ | | |
| **Hepatitis E** | ORF3 | F | 5'-attctcagcccttcgcaatc-3' | - | 89bp | |
| | | R | 5'-aacacgaggtccagcccc- 3' | - | | |
| | | Probe | 5'-aaccccttcgcccccgatgt-3' | ATTO590$^{low}$ | | |
| **External control (EC)** | | F | 5’-tgttagcaactcttcaagttccct-3’ | - | 128bp | |
| | | R | 5’-aggcaggtagggctggaaca-3’ | - | | |
| | | Probe | 5’-catggctgtccttgctcaccatcct-3’ | Cy5$^{low}$ | | |
| **INTERNAL CONTROL (HUMAN)** | B2-microglobulin | F | 5’-gcctgccgtgtgaaccatgt-3’ | - | 99bp | [4] |
| | | R | 5’-aaatgcggcatcttcaaacctc-3’ | - | | |
| | | Probe | 5’-catgatgctgcttacatgtctcgatcccac-3’ | HEX$^{low}$ | | |

**Table S2**. *In silico* designed synthetic controls that were used in the present study.

| Target | 5’→3’ Sequence |
|---|---|
| **Influenza A virus** | tcgcacagagactggaaagtgtctttgcaggaaagaacacagatcttgaggctctcatggaatggctaaagacaagaccaatcttgtcacctctgactaagggaattttaggatttgtgttcacgctcaccgtgcccagtgagcgaggactgcagcgtagacgctttatccaaaatgccctaaatggaaatggggacccg |
| **Influenza B virus** | tggccatcggatcctcaactcactcttcgagcgtctcaatgaaggacattcaaagccaattcgagcagctgaaactgcggtgggagtcttatcccaatttggtcaagagcaccgattatcaccag |
| **Respiratory syncytial virus** | atagttacaaaaaaagatggggcaaatatggaaacatacgtgaataaacttcacgagggctccacatacacagctgctgttcaatacaatgtcctagaaaaagacgatgatcctgcatcacttac |

| | |
|---|---|
| **Hepatitis A virus** | atacctcaccgccgtttgcctaggctataggctaaattttccctttccctttccctttcctattccctttgttttgcttgtaaatattaattcctgcaggttcagggttcttaaatctgtttctctataagaacactcatttttcacgctttctgtcttctttcttccagggctct |
| **Hepatitis E virus** | gcggtggtttctggggtgaccgggctgattctcagcccttcgcaatcccctatattcatccaaccaaccccttcgcccccgatgtcaccgctgcggccggggctggacctcgtgttcgccaaccc |
| **External Control** | gttgaccttaaaagtttcaaatctaggttatgttagcaactcttcaagttccctgtctcttgggggaggcattggctgaggcatgtcatagcaggtgaggtacatggctgtccttgctcaccatcctcctgagactttgttccagccctacctgcctcagaggctccggcttctcttagagaccaagag |

**Table S3.** Comparison of viral copies/µl of the newly developed multiplex RT-ddPCR assay and their corresponding singleplex assays.

| Target | Singleplex Copies/µl (SD) | Multiplex Copies/µl (SD) | p-value |
|---|---|---|---|
| **SARS-CoV-2 N1** | 13.68 (0.86) | 11.47 (0.94) | 0.10 |
| **SARS-CoV-2 N2** | 14.45 (2.39) | 11.59 (0.49) | 0.20 |
| **IAV** | 74.79 (8.51) | 65.40 (2.40) | 0.20 |
| **IBV** | 5.11 (0.13) | 11.77 (0.94) | 0.33 |
| **RSV** | 65.33 (0.80) | 60.43 (0.70) | 0.10 |
| **HAV** | 36.01 (3.30) | 34.43 (1.94) | 0.71 |
| **HEV** | 15.32 (0.37) | 16.79 (1.01) | 0.20 |
| **EC** | 94.60 (1.56) | 95.47 (2.51) | 0.75 |
| **B2M** | 5.42 (0.90) | 5.19 (0.58) | 0.70 |

**Table S4.** LOD and LOQ values obtained from the three concentration levels (CAL1, CAL2, CAL3) for the seven targets.

| Target | | Copies/µl of ddPCR | Average | SD | CV% | Copies/µl of initial sample |
|---|---|---|---|---|---|---|
| **SARS-CoV-2 N1 gene** | **CAL1** | 58.95 | 66.63 | 4.78 | 7.18 | 235.8 |
| | | 72.62 | | | | 290.5 |
| | | 65.71 | | | | 262.8 |
| | | 68.01 | | | | 272.1 |
| | | 70.08 | | | | 280.3 |
| | | 64.39 | | | | 257.6 |
| | **CAL2** | 4.44 | 5.80 | 1.12 | **19.3** | 17.8 |
| | | 4.33 | | | | 17.3 |
| | | 6.89 | | | | 27.5 |
| | | 6.22 | | | | 24.9 |
| | | 6.48 | | | | 25.9 |

| | | | | | | |
|---|---|---|---|---|---|---|
| | | 6.44 | | | | 25.8 |
| | **CAL3** | 0.77 | 0.55 | 0.25 | 46.1 | 3.1 |
| | | 0.27 | | | | 1.1 |
| | | 0.57 | | | | 2.3 |
| | | 0.19 | | | | 0.8 |
| | | 0.60 | | | | 2.4 |
| | | 0.35 | | | | 1.4 |
| | | 0.81 | | | | 3.2 |
| | | 0.36 | | | | 1.5 |
| | | 0.38 | | | | 1.5 |
| | | 0.44 | | | | 1.8 |
| | | 0.68 | | | | 2.7 |
| | | 1.28 | | | | 5.1 |
| | | 0.59 | | | | 2.3 |
| | | 0.43 | | | | 1.7 |
| | | 0.46 | | | | 1.8 |
| | | 0.45 | | | | 1.8 |
| | | 0.77 | | | | 3.1 |
| | | 0.73 | | | | 2.9 |
| | | 0.21 | | | | 0.8 |
| | | 0.64 | | | | 2.6 |
| **LOD SARS-CoV-2 N1 gene:** 2.2 copies/µl (95% CI: 1.7 copies/µl to 2.7 copies/µl)<br>**LOQ SARS-CoV-2 N1 gene:** 11.2 copies/µl<br>**Detection rate:** 20/20 | | | | | | |
| **SARS-CoV-2 N2 gene** | **CAL1** | 61.56 | 69.56 | 4.71 | 6.77 | 246.3 |
| | | 74.56 | | | | 298.3 |
| | | 71.22 | | | | 284.9 |
| | | 70.14 | | | | 280.6 |
| | | 72.97 | | | | 291.9 |
| | | 66.90 | | | | 267.6 |
| | **CAL2** | 4.44 | 6.08 | 1.18 | **19.5** | 17.8 |
| | | 5.02 | | | | 20.1 |

| | | | | | | |
|---|---|---|---|---|---|---|
| | | 7.47 | | | | 29.9 |
| | | 6.93 | | | | 27.7 |
| | | 5.83 | | | | 23.3 |
| | | 6.78 | | | | 27.1 |
| | **CAL3** | 0.39 | 0.50 | 0.21 | 41.7 | 1.5 |
| | | 0.20 | | | | 0.8 |
| | | 0.29 | | | | 1.1 |
| | | 0.19 | | | | 0.8 |
| | | 0.37 | | | | 1.5 |
| | | 0.43 | | | | 1.7 |
| | | 0.50 | | | | 2.0 |
| | | 0.36 | | | | 1.5 |
| | | 0.58 | | | | 2.3 |
| | | 0.51 | | | | 2.0 |
| | | 0.41 | | | | 1.6 |
| | | 0.99 | | | | 4.0 |
| | | 0.80 | | | | 3.2 |
| | | 0.43 | | | | 1.7 |
| | | 0.65 | | | | 2.6 |
| | | 0.65 | | | | 2.6 |
| | | 0.64 | | | | 2.6 |
| | | 0.40 | | | | 1.6 |
| | | 0.35 | | | | 1.4 |
| | | 0.79 | | | | 3.1 |
| **LOD SARS-CoV-2 N2 gene:** 2.0 copies/µl (95% CI: 1.6 copies/µl to 2.4 copies/µl)<br>**LOQ SARS-CoV-2 N2 gene:** 11.8 copies/µl<br>**Detection rate:** 20/20 | | | | | | |
| **IAV** | **CAL1** | 38.90 | 40.21 | 2.71 | 6.75 | 155.6 |
| | | 43.34 | | | | 173.3 |
| | | 43.19 | | | | 172.8 |
| | | 40.51 | | | | 162.1 |
| | | 38.95 | | | | 155.8 |

| | | | | | | |
|---|---|---|---|---|---|---|
| | | 36.37 | | | | 145.5 |
| | **CAL2** | 3.33 | 3.46 | 0.80 | **23.2** | 13.3 |
| | | 4.33 | | | | 17.3 |
| | | 2.12 | | | | 8.5 |
| | | 3.62 | | | | 14.5 |
| | | 4.19 | | | | 16.8 |
| | | 3.15 | | | | 12.6 |
| | **CAL3** | 0.19 | 0.36 | 0.16 | 43.7 | 0.8 |
| | | 0.54 | | | | 2.2 |
| | | 0.14 | | | | 0.6 |
| | | 0.52 | | | | 2.1 |
| | | 0.30 | | | | 1.2 |
| | | 0.21 | | | | 0.9 |
| | | 0.62 | | | | 2.5 |
| | | 0.61 | | | | 2.4 |
| | | 0.51 | | | | 2.0 |
| | | 0.32 | | | | 1.3 |
| | | [1]ND | | | | 0.3 |
| | | [1]ND | | | | 0.3 |
| | | 0.37 | | | | 1.5 |
| | | 0.31 | | | | 1.2 |
| | | 0.20 | | | | 0.8 |
| | | 0.26 | | | | 1.0 |
| | | 0.19 | | | | 0.8 |
| | | 0.53 | | | | 2.1 |
| | | 0.35 | | | | 1.4 |
| | | 0.29 | | | | 1.1 |
| **LOD IAV:** 1.4 copies/µl (95% CI: 1.1 copies/µl to 1.7 copies/µl)<br>**LOQ IAV:** 8.0 copies/µl<br>**Detection rate:** 18/20 | | | | | | |
| **IBV** | **CAL1** | 71.92 | 72.79 | 4.05 | 5.57 | 287.7 |
| | | 74.29 | | | | 297.1 |

| | | | | | | |
|---|---|---|---|---|---|---|
| | | 68.56 | | | | 274.2 |
| | | 78.84 | | | | 315.3 |
| | | 68.28 | | | | 273.1 |
| | | 74.85 | | | | 299.4 |
| | **CAL2** | 5.82 | 5.45 | 0.69 | **12.6** | 23.3 |
| | | 4.90 | | | | 19.6 |
| | | 6.00 | | | | 24.0 |
| | | 4.53 | | | | 18.1 |
| | | 6.29 | | | | 25.2 |
| | | 5.16 | | | | 20.7 |
| | **CAL3** | 0.52 | 0.51 | 0.20 | 38.1 | 2.1 |
| | | 0.95 | | | | 3.8 |
| | | 0.57 | | | | 2.3 |
| | | 0.58 | | | | 2.3 |
| | | 0.52 | | | | 2.1 |
| | | 0.64 | | | | 2.6 |
| | | 0.69 | | | | 2.7 |
| | | 0.67 | | | | 2.7 |
| | | 0.70 | | | | 2.8 |
| | | 0.32 | | | | 1.3 |
| | | 0.27 | | | | 1.1 |
| | | 0.64 | | | | 2.5 |
| | | 0.66 | | | | 2.6 |
| | | 0.24 | | | | 1.0 |
| | | 0.33 | | | | 1.3 |
| | | 0.45 | | | | 1.8 |
| | | 0.19 | | | | 0.8 |
| | | 0.33 | | | | 1.3 |
| | | 0.62 | | | | 2.5 |
| | | 0.36 | | | | 1.4 |

**LOD IBV:** 2.1 copies/µl (95% CI: 1.7 copies/µl to 2.4 copies/µl)
**LOQ IBV:** 6.9 copies/µl

| **Detection rate:** 20/20 | | | | | | |
|---|---|---|---|---|---|---|
| **RSV** | **CAL1** | 68.70 | 69.94 | 3.10 | 4.43 | 274.8 |
| | | 71.36 | | | | 285.5 |
| | | 69.76 | | | | 279.0 |
| | | 74.69 | | | | 298.8 |
| | | 69.87 | | | | 279.5 |
| | | 65.27 | | | | 261.1 |
| | **CAL2** | 5.36 | 5.89 | 0.52 | **8.87** | 21.4 |
| | | 6.22 | | | | 24.9 |
| | | 6.67 | | | | 26.7 |
| | | 5.31 | | | | 21.2 |
| | | 6.03 | | | | 24.1 |
| | | 5.77 | | | | 23.1 |
| | **CAL3** | 0.65 | 0.72 | 0.22 | 31.1 | 2.6 |
| | | 0.95 | | | | 3.8 |
| | | 0.50 | | | | 2.0 |
| | | 0.45 | | | | 1.8 |
| | | 0.67 | | | | 2.7 |
| | | 1.13 | | | | 4.5 |
| | | 0.87 | | | | 3.5 |
| | | 0.55 | | | | 2.2 |
| | | 0.70 | | | | 2.8 |
| | | 0.82 | | | | 3.3 |
| | | 0.81 | | | | 3.2 |
| | | 1.28 | | | | 5.1 |
| | | 0.73 | | | | 2.9 |
| | | 0.67 | | | | 2.7 |
| | | 0.65 | | | | 2.6 |
| | | [1]ND | | | | 0.0 |
| | | 0.64 | | | | 2.6 |
| | | 0.60 | | | | 2.4 |

| | | | | | | |
|---|---|---|---|---|---|---|
| | | 0.62 | | | | 2.5 |
| | | 0.36 | | | | 1.4 |

**LOD RSV:** 2.9 copies/µl (95% CI: 2.4 copies/µl to 3.3 copies/µl)
**LOQ RSV:** 5.2 copies/µl
**Detection rate:** 19/20

| | | | | | | |
|---|---|---|---|---|---|---|
| **HAV** | **CAL1** | 60.11 | 63.29 | 3.01 | 4.75 | 240.5 |
| | | 68.45 | | | | 273.8 |
| | | 63.56 | | | | 254.2 |
| | | 63.90 | | | | 255.6 |
| | | 60.45 | | | | 241.8 |
| | | 63.29 | | | | 253.2 |
| | **CAL2** | 5.88 | 6.14 | 0.83 | **13.5** | 23.5 |
| | | 5.72 | | | | 22.9 |
| | | 5.34 | | | | 21.4 |
| | | 6.93 | | | | 27.7 |
| | | 7.40 | | | | 29.6 |
| | | 5.57 | | | | 22.3 |
| | **CAL3** | 0.65 | 0.45 | 0.18 | 40.8 | 2.6 |
| | | 0.27 | | | | 1.1 |
| | | 0.79 | | | | 3.2 |
| | | 0.39 | | | | 1.6 |
| | | [1]ND | | | | |
| | | [1]ND | | | | |
| | | 0.50 | | | | 2.0 |
| | | 0.61 | | | | 2.4 |
| | | 0.38 | | | | 1.5 |
| | | 0.45 | | | | 1.8 |
| | | 0.54 | | | | 2.2 |
| | | 0.85 | | | | 3.4 |
| | | 0.22 | | | | 0.9 |
| | | 0.24 | | | | 1.0 |
| | | 0.39 | | | | 1.6 |

| | | | | | | |
|---|---|---|---|---|---|---|
| | | 0.19 | | | | 0.8 |
| | | 0.45 | | | | 1.8 |
| | | 0.47 | | | | 1.9 |
| | | 0.35 | | | | 1.4 |
| | | 0.36 | | | | 1.4 |
| **LOD HAV:** 1.8 copies/µl (95% CI: 1.4 copies/µl to 2.2 copies/µl)<br>**LOQ HAV:** 8.3 copies/µl<br>**Detection rate:** 18/20 | | | | | | |
| **HEV** | **CAL1** | 64.04 | 66.90 | 2.96 | 4.42 | 256.1 |
| | | 69.14 | | | | 276.6 |
| | | 70.14 | | | | 280.6 |
| | | 69.32 | | | | 277.3 |
| | | 65.26 | | | | 261.0 |
| | | 63.52 | | | | 254.1 |
| | **CAL2** | 4.70 | 4.61 | 0.45 | **9.84** | 18.8 |
| | | 5.02 | | | | 20.1 |
| | | 4.83 | | | | 19.3 |
| | | 4.85 | | | | 19.4 |
| | | 4.52 | | | | 18.1 |
| | | 3.75 | | | | 15.0 |
| | **CAL3** | 0.58 | 0.48 | 0.15 | 31.4 | 2.3 |
| | | 0.34 | | | | 1.4 |
| | | 0.57 | | | | 2.3 |
| | | 0.19 | | | | 0.8 |
| | | 0.30 | | | | 1.2 |
| | | 0.43 | | | | 1.7 |
| | | 0.50 | | | | 2.0 |
| | | 0.42 | | | | 1.7 |
| | | 0.58 | | | | 2.3 |
| | | 0.57 | | | | 2.3 |
| | | 0.61 | | | | 2.4 |
| | | 0.42 | | | | 1.7 |

| | | | | | | |
|---|---|---|---|---|---|---|
| | | 0.66 | | | | 2.6 |
| | | 0.24 | | | | 1.0 |
| | | 0.52 | | | | 2.1 |
| | | 0.58 | | | | 2.3 |
| | | 0.51 | | | | 2.0 |
| | | 0.53 | | | | 2.1 |
| | | 0.28 | | | | 1.1 |
| | | 0.79 | | | | 3.1 |
| **LOD HEV:** 1.9 copies/µl (95% CI: 1.6 copies/µl to 2.2 copies/µl)<br>**LOQ HEV:** 4.5 copies/µl<br>**Detection rate:** 20/20 | | | | | | |

[1]ND: Non-Detected
The lowest detected concentration with a CV ≤ 25 (LOQ) is represented in bold.

**Table S5.** Copies/L of N1 and N2 targets of SARS-CoV-2 for each sample.

| SAMPLE | Sampling Date (day-month-year) | SARS-CoV-2 N1 (copies/L) | SARS-CoV-2 N2 (copies/L) | Average (copies/L) |
|---|---|---|---|---|
| **#1** | **23/12/2023** | 1.22E+06 | 1.28E+06 | 1.25E+06 |
| **#2** | **25/12/2023** | 2.20E+06 | 2.31E+06 | 2.25E+06 |
| **#3** | **28/12/2023** | 1.67E+06 | 1.74E+06 | 1.71E+06 |
| **#4** | **15/2/2023** | 1.10E+05 | 1.20E+05 | 1.15E+05 |
| **#5** | **18/2/2024** | 1.31E+05 | 1.47E+05 | 1.39E+05 |
| **#6** | **5/3/2023** | 4.60E+04 | 4.94E+04 | 4.77E+04 |
| **#7** | **7/3/2024** | 5.08E+04 | 5.58E+04 | 5.33E+04 |
| **#8** | **4/4/2024** | 4.53E+04 | 4.95E+04 | 4.74E+04 |
| **#9** | **11/4/2024** | 7.15E+04 | 7.08E+04 | 7.12E+04 |
| **#10** | **3/5/2024** | 4.80E+04 | 5.93E+04 | 5.37E+04 |
| **#11** | **7/5/2024** | 9.40E+04 | 9.87E+04 | 9.64E+04 |
| **#12** | **9/5/2024** | 1.13E+05 | 1.32E+05 | 1.23E+05 |
| **#13** | **4/11/2024** | 2.20E+05 | 2.24E+05 | 2.22E+05 |
| **#14** | **6/11/2024** | 3.03E+05 | 2.84E+05 | 2.94E+05 |
| **#15** | **8/11/2024** | 7.36E+04 | 9.00E+04 | 8.18E+04 |
| **#16** | **10/11/2024** | 4.21E+05 | 3.99E+05 | 4.10E+05 |
| **#17** | **21/11/2024** | 3.29E+05 | 3.19E+05 | 3.24E+05 |

| | | | | |
|---|---|---|---|---|
| **#18** | **3/12/2024** | 3.83E+05 | 3.80E+05 | 3.81E+05 |
| **#19** | **8/12/2024** | 3.49E+05 | 3.50E+05 | 3.49E+05 |
| **#20** | **24/12/2024** | 7.02E+05 | 6.85E+05 | 6.94E+05 |
| **#21** | **27/12/2024** | 4.44E+05 | 4.96E+05 | 4.70E+05 |
| **#22** | **29/12/2024** | 6.81E+05 | 6.10E+05 | 6.46E+05 |
| **#23** | **1/1/2025** | 5.28E+05 | 5.35E+05 | 5.32E+05 |
| **#24** | **3/1/2025** | 5.62E+05 | 5.58E+05 | 5.60E+05 |
| **#25** | **6/1/2025** | 6.85E+05 | 6.62E+05 | 6.73E+05 |
| **#26** | **17/1/2025** | 1.94E+05 | 2.11E+05 | 2.02E+05 |
| **#27** | **21/1/2025** | 1.63E+05 | 1.61E+05 | 1.62E+05 |
| **#28** | **23/1/2025** | 2.23E+05 | 2.13E+05 | 2.18E+05 |
| **#29** | **25/1/2025** | 1.90E+05 | 1.64E+05 | 1.77E+05 |
| **#30** | **30/1/2025** | 1.50E+05 | 1.57E+05 | 1.53E+05 |
| **#31** | **4/2/2025** | 1.06E+05 | 1.13E+05 | 1.10E+05 |
| **#32** | **6/2/2025** | 9.85E+04 | 1.14E+05 | 1.06E+05 |
| **#33** | **8/2/2025** | 1.30E+05 | 1.22E+05 | 1.26E+05 |
| **#34** | **10/2/2025** | 1.18E+05 | 1.07E+05 | 1.12E+05 |
| **#35** | **13/2/2025** | 1.00E+05 | 1.09E+05 | 1.05E+05 |
| **#36** | **15/2/2025** | 7.66E+04 | 8.35E+04 | 8.01E+04 |
| **#37** | **18/2/2025** | 7.74E+04 | 8.81E+04 | 8.28E+04 |
| **#38** | **23/2/2025** | 9.54E+04 | 8.76E+04 | 9.15E+04 |

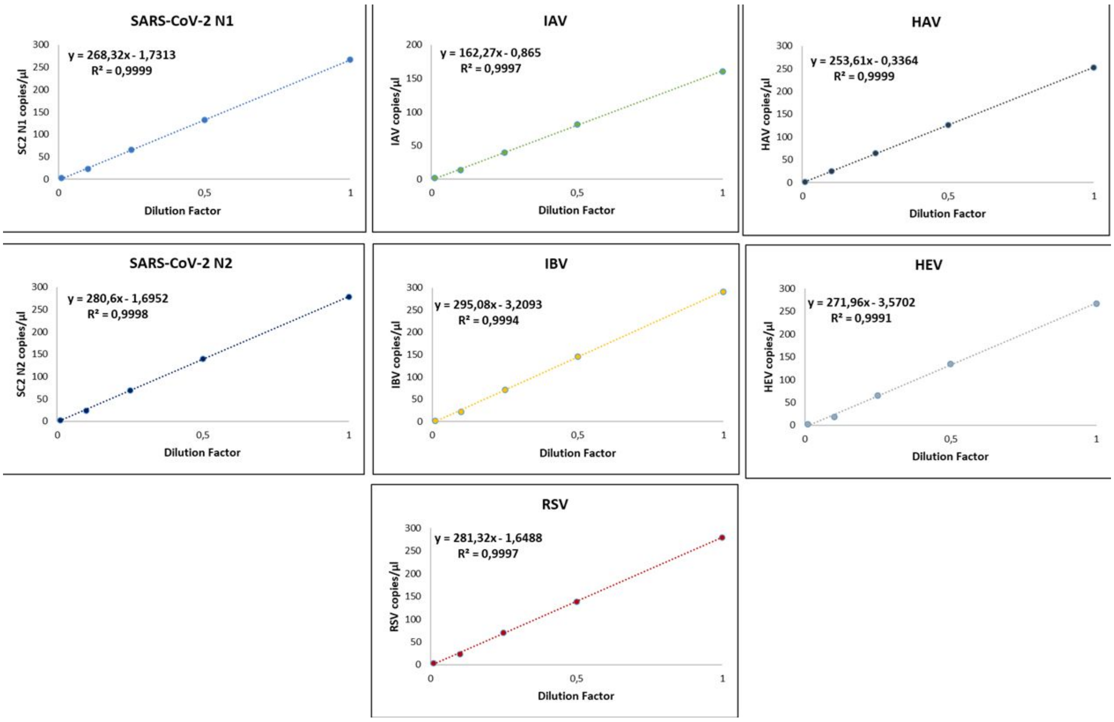


**Figure S1.** Linear dynamic range of the developed RT-ddPCR assay for the targeted viruses. SC2; SARS-CoV-2, IAV; Influenza A virus, IBV; Influenza B virus, RSV; Respiratory Syncytial Virus, HAV; Hepatitis A virus, HEV; Hepatitis E virus.

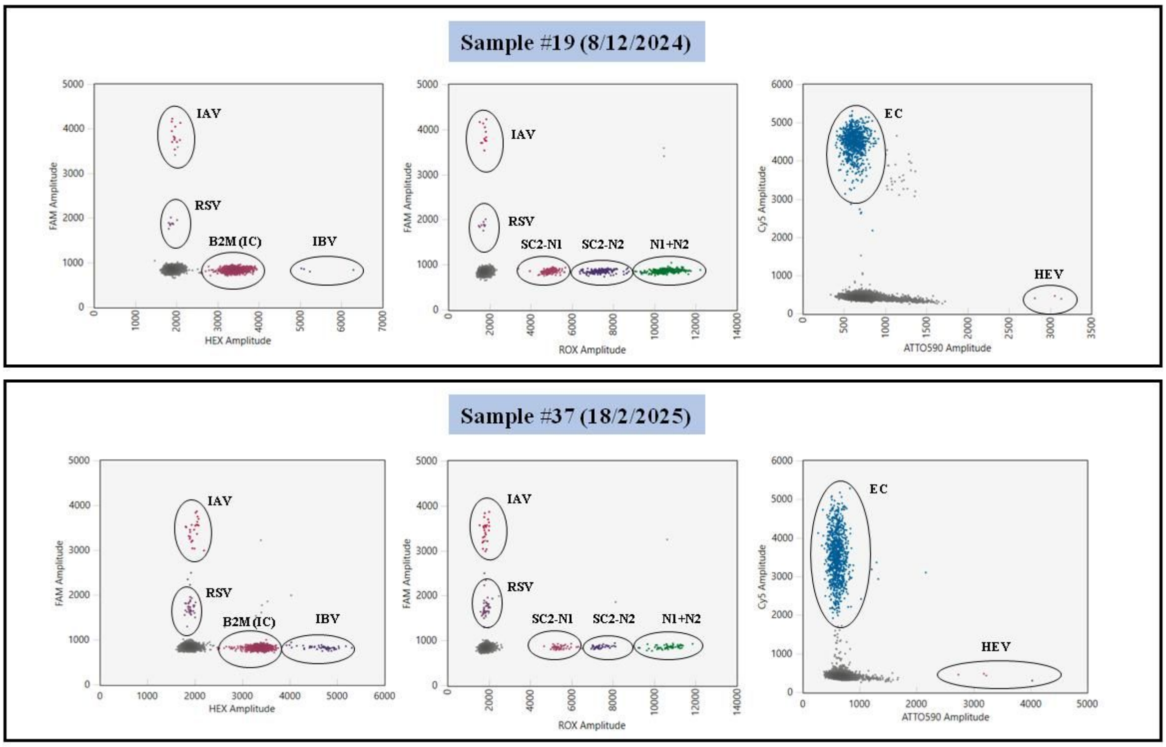


**Figure S2.** Representative RT-ddPCR dot plots from two wastewater samples.

**References**

(1) Shu, B.; Kirby, M. K.; Davis, W. G.; Warnes, C.; Liddell, J.; Liu, J.; Wu, K. H.; Hassell, N.; Benitez, A. J.; Wilson, M. M.; Keller, M. W.; Rambo-Martin, B. L.; Camara, Y.; Winter, J.; Kondor, R. J.; Zhou, B.; Spies, S.; Rose, L. E.; Winchell, J. M.; Limbago, B. M.; Wentworth, D. E.; Barnes, J. R. Multiplex Real-Time Reverse Transcription PCR for Influenza A Virus, Influenza B Virus, and Severe Acute Respiratory Syndrome Coronavirus 2 - Volume 27, Number 7—July 2021 - Emerging Infectious Diseases Journal - CDC. *Emerg. Infect. Dis.* **2021**, *27* (7), 1821–1830. https://doi.org/10.3201/EID2707.210462.

(2) Fry, A. M.; Chittaganpitch, M.; Baggett, H. C.; Peret, T. C. T.; Dare, R. K.; Sawatwong, P.; Thamthitiwat, S.; Areerat, P.; Sanasuttipun, W.; Fischer, J.; Maloney, S. A.; Erdman, D. D.; Olsen, S. J. The Burden of Hospitalized Lower Respiratory Tract Infection Due to Respiratory Syncytial Virus in Rural Thailand. *PLoS One* **2010**, *5* (11), e15098. https://doi.org/10.1371/JOURNAL.PONE.0015098.

(3) Lu, X.; Wang, L.; Sakthivel, S. K.; Whitaker, B.; Murray, J.; Kamili, S.; Lynch, B.; Malapati, L.; Burke, S. A.; Harcourt, J.; Tamin, A.; Thornburg, N. J.; Villanueva, J. M.; Lindstrom, S. US CDC Real-Time Reverse Transcription PCR Panel for Detection of Severe Acute Respiratory Syndrome Coronavirus 2. *Emerg. Infect. Dis.* **2020**, *26* (8), 1654. https://doi.org/10.3201/EID2608.201246.

(4) Strati, A.; Lianidou, E. S.; Zavridou, M.; Paraskevis, D.; Magiorkinis, G.; Sapounas, S.; Lagiou, P.; Thomaidis, N. S. Development and Analytical Validation of a One-Step Five-Plex RT-DdPCR Assay for the Quantification of SARS-CoV-2 Transcripts in Clinical Samples. *Anal. Chem.* **2022**, *94* (36), 12314–12322. https://doi.org/10.1021/ACS.ANALCHEM.2C00868.
